# Suitable hepatitis B vaccine for adult immunization in China

**DOI:** 10.1007/s12026-015-8742-1

**Published:** 2015-12-08

**Authors:** Linna Yang, Jun Yao, Jing Li, Yongdi Chen, Zheng-gang Jiang, Jing-jing Ren, Kai-jin Xu, Bing Ruan, Shi-gui Yang, Bing Wang, Tian-sheng Xie, Qian Li

**Affiliations:** School of Medicine, Ningbo University, Ningbo, 315211 China; Zhejiang Provincial Center for Disease Control and Prevention, Hangzhou, 310051 Zhejiang China; Zhejiang Provincial Hospital, Hangzhou, 310013 Zhejiang China; State Key Laboratory for Diagnosis and Treatment of Infectious Disease, Key Laboratory of Infectious Diseases, First Affiliated Hospital, School of Medicine, Zhejiang University, Hangzhou, 310003 China

**Keywords:** Adults, Hepatitis B vaccine, Vaccine immunogenicity

## Abstract

The aim of this study was to evaluate, in adults, the immunogenicity of six hepatitis B vaccines with different doses or different manufacturers in the Chinese market and to provide evidence to support adult hepatitis B vaccination. Participants were randomly divided into six groups (I–VI). Six vaccines (4 at 10 μg/dose and 2 at 20 μg/dose) were administered intramuscularly to healthy adults at 0, 1 and 6 month intervals. All participants (16–50 years) who were negative for any hepatitis B virus serological markers were vaccinated. Anti-HBs levels were assessed 1 month and 1 year after the third vaccination. The anti-HBs seroconversion rate (anti-HBs >10mIU/ml) was 99.4 % (99.9 % for 10 μg dose groups and 97.9 % for 20 μg dose groups) 1 month after the third vaccination, and the anti-HBs seroreversion rate was 77.0 % (75.3 and 82.6 %) 1 year after the third vaccination (*n* = 1036). One month after completing the vaccinations, the seroconversion rates were not significantly different (100.0, 100.0, 99.6, 100.0 %) for the four 10 μg dose and two 20 μg dose groups (99.1, 96.9 %). One year after the third vaccination, the group II positive rate was significantly higher than the other three 10 μg dose groups, and the group VI positive rate was significantly higher than the other 20 μg dose group. Groups II and VI showed a significantly higher positive rate and anti-HBs geometric mean titer (GMT) than the other groups. The anti-HBs level declined with increasing age, and the seroreversion rate and GMT decreased over time. All six vaccines had high anti-HBs seroconversion rates and good immunization effects. The 10 μg dose vaccine (Dalian High-Tech) and the 20 μg dose vaccine (GlaxoSmithKline) are recommended for adults.

## Introduction

Hepatitis B virus (HBV) infection is a current public health problem worldwide [1]. Approximately 780,000 people die each year because of this infection, and more than 240 million have chronic HBV infection, which also serves as the main reservoir for continued HBV transmission [2]. HBV infection is especially severe in China. According to a national serum epidemiological survey conducted in 2006, the rate of HBsAg carriers is 7.18 % in the general population within the age range of 1–59 years [3, 4]. Based on this prevalence, over 93 million people are infected with chronic HBV in China [5]. The hepatitis B vaccine was the first vaccine to prevent a chronic disease and a sexually transmitted infection. Hepatitis B vaccination is regarded as the most economical and effective method for preventing and controlling hepatitis B infection because there is no satisfactory treatment for chronic hepatitis B infection and related diseases [6–8].

Hepatitis B vaccine has been part of planned immunization management in China since 1992. The hepatitis B infection rate and morbidity in children have decreased significantly with routine neonatal vaccination, but it has not been as successful in adults. Adult hepatitis B immunization in China needs further support. Because adult hepatitis B vaccination has not been systematically carried out in many regions, there are insufficient immunological performance data for the hepatitis B vaccine. To assist with the advancement of the hepatitis B vaccination and control the process in adults, we studied the effects of six different hepatitis B vaccines that are common in the Chinese market: 10 or 20 μg dose vaccines from four companies. Adults were given a 10 or 20 μg dose of one hepatitis B vaccine at 0-, 1- and 6-month intervals. The immunogenicity of all six hepatitis B vaccines was evaluated at 1 month and 1 year after completing the vaccination schedule.

## Materials and methods

### Study participants

The study was conducted in seven counties (Deqing, Changxing, Anji, Nanxun, Wuxing, Shaoxing and Tongxiang) in the Zhejiang Province, China. The economic status of the selected counties was similar. The present study was approved by the ethics committee. All participants were willing to participate in the study and they each provided written informed consent before any study-related procedures were performed. We used the questionnaire “Research Questionnaire on Adult Immunization Strategy of Hepatitis B,” which contained basic information such as each participant’s birth date, age and gender. We collected 3-ml blood samples from each subject prior to vaccination. The first blood draw and the first shot were carried out simultaneously (inoculation after the blood draw). Eligible subjects received three hepatitis B vaccinations, and we collected 3-ml blood samples from each subject at 1 month (210 days from the first dose) and 1 year (545 days from the first dose) after the third vaccination, which were preserved for anti-HBs quantification. Only those participants who were negative for HBsAg, anti-HBs and anti-HBc were analyzed in the study and these participants were divided into six groups, I to VI, based on the different vaccine types. The study was approved by the Institutional Ethics Committee at the Zhejiang Center for Disease Control and Prevention, China. Specific inclusion and exclusion criteria are as follows:

*Inclusion criteria* (a) age 16–50 years and willing to participate in the study and sign the informed consent form; (b) willing to participate in the follow-up study and to provide the one-month and one-year blood samples after the third dose.

*Exclusion criteria* (a) reluctant to participate in this study; (b) HBsAg positive and/or anti-HBs positive; (c) history of allergies or severe reaction to vaccination; (d) history of hepatitis B vaccination; (e) history of any kind of vaccination within the previous 4 weeks; (f) high risk of becoming immunologically compromised; (g) previous immune suppressive therapy (intravenous or oral cortisone or chemotherapy); (h) previous immunostimulation therapy; (i) received any kind of observational or experimental drugs during the past 4 weeks; (j) acute illness within the past 7 days; (k) infection that required treatment with antibacterial or antiviral therapy within the past 7 days; (l) fever within the past 3 days (subaxillary temperature ≥38 °C); (m) known or anticipated immune dysfunction.

### Vaccines and vaccination

The hepatitis B vaccines used in our study were common vaccines in the Chinese market. Subjects were assigned to one of six groups based on the vaccine type: (1) group I: hepatitis B vaccine (lot No. 20090521; dose: 10 μg; Shenzhen Kangtai Biological Products Co., Ltd., China); (2) group II: hepatitis B vaccine (lot Nos. 2009030906 and 2010010106; dose: 10 μg; Dalian High-Tech Biopharmaceutical Co., Ltd., China); (3) group III: hepatitis B vaccine (lot No. 200904A3101; dose: 10 μg; North China Pharmaceutical Company, GeneTech Bio-technology Pharmaceutical Co., Ltd; Chinese Hamster Ovary (CHO)); (4) group IV, hepatitis B vaccine (lot No. XHBVB554AA; dose: 10 μg; GlaxoSmithKline, UK); (5) group V, hepatitis B vaccine (lot No. 200904A3101; dose: 20 μg; North China Pharmaceutical Company, GeneTech Bio-technology Pharmaceutical Co., Ltd; CHO); and (6) group VI, hepatitis B vaccine (lot No. XHBVB554AA; dose: 20 μg; GlaxoSmithKline, UK). Each vaccine was administered intramuscularly in the upper deltoid muscle, according to the recommended immunization procedure, at 0, 1 (30 days from the first dose) and 6 (180 days from the first dose) months.

### Lab testing

#### Sample collection and processing

Blood samples (3 ml) were collected before vaccination from each qualified participant and at 1 and 12 months after the third vaccination. The samples from each participant were preserved at −20 °C for later analysis. All participants remained at the clinic for 30 min after each injection, to watch for immediate adverse reactions. Frozen separated serum samples were sent to ADICON Clinical Laboratories, Inc. (Hangzhou) for quantification of HBsAg, anti-HBs and anti-HBc using chemiluminescence immunoassay (CLIA).

#### Apparatus and reagents

We used an Architect-i2000 (Abbot, US) to perform the chemical luminescence immunoassay. The reagent lot number for HBsAg tests was 70318HN00, with the criterion that a signal-to-noise ratio (S/N) ≥0.05 is considered to be positive. The reagent lot number for the anti-HBs test was 75684 M100, with the criterion that an anti-HBs antibody level equal to or higher than 10 mIU/ml was regarded as being positive and was considered to have protective effects against HBV infection; an anti-HBs antibody level equal to or higher than 100 mIU/ml was defined as a good response. The reagent lot number for the anti-HBc test was 72448 M100, with the criterion that an anti-HBc antibody level equal to or higher than 1 mIU/ml was defined as positive.

### Data collation and analysis

We established a database using EpiData3.2 (EpiData; Norway and Denmark), and statistical analysis was performed using SPSS 18.0 and Excel 2007. Chi-square test or Fisher’s exact test was used for enumeration data and analysis of variance was used for measurement data. The relationship between anti-HBs level and the age and anti-HBs levels at two time points was compared using a bivariable correlation test. The interactions between age, sex and vaccine were performed using univariate analysis of variance. A two-tailed probability was used in statistical tests, and *α* = 0.05 was considered to be significant. The data of antibody titer were normal distribution through a logarithmic conversion.

## Results

### Study subject characteristics

**Fig. 1:**
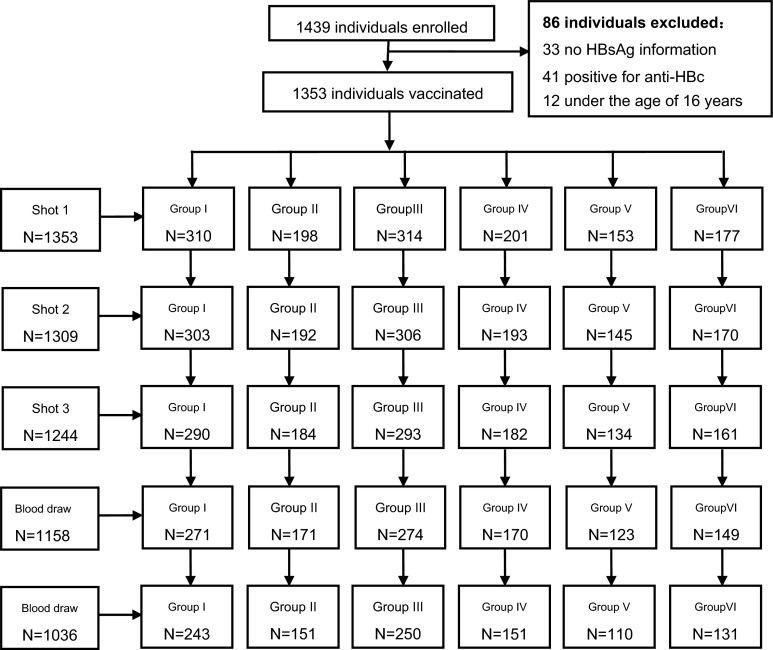
Flow chart of the participants enrolled in the study

A total of 1439 individuals were enrolled and received the vaccination between March and June 2010, and blood samples were collected at 1 month and 1 year after the final vaccination. There were 403 individuals excluded: 33 subjects had no HBsAg information, 41 subjects were positive for anti-HBc, 12 subjects were under the age of 16 years and 317 subjects were lost to follow-up (Fig. 1). Data from a total of 1036 subjects were analyzed in this study; there were 420 male and 616 female subjects without any reactive hepatitis B serological markers. The average subject age was 32.64 years (range 16.02–49.16 years). There were 243, 151, 250, 110, 131 and 151 subjects in the six groups (I–VI), respectively. There were no statistically significant differences in age or gender between the six groups (Table 1).

**Table 1:** Age and sex distribution of study subjects

Vaccine group	Gender		*χ* ^2^	*p*	Age				*χ* ^*2*^	*p*
	Male	Female			15–24	25–34	35–44	45–50		
I	99	144	6.757	0.239	37	97	88	21	22.048	0.107
II	50	101			19	62	56	14		
III	107	143			48	106	77	19		
IV	65	86			40	62	39	10		
V	51	59			21	52	25	12		
VI	48	83			31	49	41	10		

### Antibody response in the two different vaccine dose groups

There were 1036 subjects who achieved immune response, with a total seroconversion rate of 99.4 % and an anti-HBs geometric mean titer (GMT) of 429.06 mIU/ml (95 % CI 381.07–483.10) at 1 month after the third vaccination, and a total positive rate of 77.0 % and an anti-HBs GMT of 47.36 mIU/ml (95 % CI 41.10–54.58) at 1 year after the third vaccination. Both the anti-HBs seroreversion rate and the anti-HBs GMT decreased obviously at 1 year after the third vaccination. There were statistical differences in the seroconversion rates between the 10 μg dose vaccine group and 20 μg dose vaccine group at 1 month and in the seroreversion rates at 1 year after the third vaccination (*p* = 0.003 and *p* = 0.019, respectively). The seroconversion rate in the 10 μg dose group was higher than that of 20 μg dose group (99.9 and 97.9 %, respectively) at 1 month after the third vaccination, but the positive rate in the 20 μg dose group was higher than that of 10 μg dose group at 1 year after the third vaccination (82.6 % and 75.3 %, respectively). The anti-HBs GMTs at the two time points were 429.06 mIU/ml (414.28 mIU/ml for the 10 μg dose group and 481.64 mIU/ml for the 20 μg dose group) and 47.36 mIU/ml (46.59 mIU/ml for the 10 μg dose group and 50.00 mIU/ml for 20 μg dose group). The differences in the anti-HBs GMTs between the 10 μg dose group and the 20 μg dose group were not statistically significant at the two time points (*p* = 0.293 and *p* = 0.680, respectively).

### Comparison of the immunization effects of different 10 μg dose vaccines

**Fig. 2:**
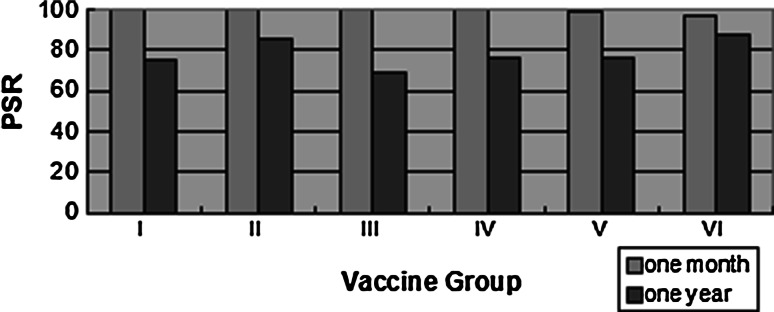
Positive seroconversion rate (PSR) of different vaccines

The seroconversion rates for the four 10 μg dose vaccine groups were 100.0, 100.0, 99.6 and 100.0 %, respectively, and the differences in the seroconversion rates between the four groups were not statically significant (*p* = 1.000, Fisher’s exact test) at 1 month after the third vaccination. The positive rates of the four groups were 74.9, 85.4, 69.2 and 76.2 %, respectively, at 1 year after the third vaccination, and the positive rate for group II was significantly higher than the other three groups (*p* = 0.004); group II also showed the least anti-HBs positive proportion decrease over time. The anti-HBs GMTs were 414.28 mIU/ml (95 % CI 364.88–470.38) and 46.59 mIU/ml (95 % CI 39.30–55.23) at the two time points. The anti-HBs GMTs of the four groups were significantly different at the two time points (*p* < 0.001), and the anti-HBs GMTs of group II were the highest at two time points though it had great decline at 1 year. The anti-HBs GMTs of the four groups were 304.11, 906.07, 330.33 and 453.25 mIU/ml at 1 month after the third vaccination, and 33.60, 98.30, 34.42 and 61.69 mIU/ml at 1 year after the third vaccination (Table 2; Fig. 2).

**Table 2:** Anti-HBs PSR and GMT of different dose vaccines at two time points

Vaccine group	*N*	*n*		PSR (%)		GMT		95 % CI	
		One month	One year	One month	One year	One month	One year	One month	One year
I	243	243	182	100.0	74.9	304.11	33.60	244.15–378.78	24.36–46.34
II	151	151	129	100.0	85.4	906.07	98.30	693.63–1183.57	70.18–130.14
III	250	249	173	99.6	69.2	330.33	34.42	263.61–413.95	25.66–46.17
IV	151	151	115	100.0	76.2	453.25	61.69	333.10–616.73	41.32–92.12
Total	795	794	599	99.9	75.3	414.28	46.59	364.88–470.38	39.30–55.23
*χ* ^*2*^ */F*					13.430	13.617	8.313		
*P*				1.00^a^	0.004	<0.001	<0.001		
V	110	109	84	99.1	76.4	142.98	33.28	107.43–190.29	22.93–48.31
VI	131	127	115	96.9	87.8	1335.45	70.37	888.05–2008.24	51.89–95.44
Total	241	236	199	97.9	82.6	481.64	50.00	359.62–645.07	39.32–63.58
*χ* ^*2*^ */F*					5.421	73.267	9.685		
*P*				0.379^a^	0.026	<0.001	0.002		

^a^Fisher’s exact test

### Comparison of immunization effects of different 20 μg dose vaccines

The seroconversion rates of the two 20 μg dose groups were 99.1 and 96.9 %, and the difference was not significant (*p* = 0.379) at 1 month after the third vaccination. The positive rate in group VI was significantly higher than that of group V (87.8 and 76.4 %, respectively, *p* = 0.026) at 1 year after the third vaccination. The anti-HBs GMTs were 481.64 mIU/ml (95 % CI 359.62–645.07) and 50.00 mIU/ml (95 % CI 39.32–63.58) at the two time points. The anti-HBs GMTs in two groups were significantly different at the two time points (*p* < 0.001 and *p* < 0.002, respectively), and the anti-HBs GMTs in group VI were higher than that of group V at 1 month and 1 year after the third vaccination, although there was a large reduction in the anti-HBs GMTs at the second time point. The anti-HBs GMTs in the two groups were 142.98 and 1335.45 mIU/ml at 1 month after the third vaccination, and 33.28 and 70.37 mIU/ml at 1 year after the third vaccination (Table 2; Fig. 2).

### Comparison of the immunization effects in different gender groups after vaccination

All six vaccines achieved high seroconversion rates in both males and females at 1 month after the third vaccination, and differences in anti-HBs rates for the six vaccines were not statistically significant between the genders (*p* = 0.428, Fisher’s exact test, *p* = 0.464, *p* = 0.139). There were also no statistical differences between anti-HBs positive rates in males and females in the six groups at 1 year after the third vaccination (*p* = 0.343, *p* = 0.183, *p* = 0.571, *p* = 0.848, *p* = 0.670, *p* = 0.237).

The anti-HBs GMT in the female group was higher than that of male group at 1 month after the full vaccination: 505.02 and 337.77 mIU/ml, respectively, and the difference was significant (*p* = 0.001). Only in group II was the anti-HBs GMT statistically different between males and females at the two time points (*p* = 0.001 and *p* = 0.003, respectively), and the anti-HBs GMTs in the female group were higher than those in the male group: 1239.90 and 142.43 mIU/ml for females, and 480.82 and 46.48 mIU/ml for males. The anti-HBs GMT for females in group V was significantly higher than that of males at 1 month after the final vaccination (*p* = 0.036): 189.30 mIU/ml for females and 103.34 mIU/ml for males (Table 3).

**Table 3:** Anti-HBs GMT in different gender groups after vaccination at two time points

Gender	Group I		Group II		Group III		Group IV		Group V		Group VI		Total	
	*n*	GMT	*n*	GMT	*n*	GMT	*n*	GMT	*n*	GMT	*n*	GMT	*n*	GMT
*One month*														
Male	99	304.54	50	480.82	107	294.52	65	463.39	51	103.34	48	901.29	420	337.77
Female	144	303.81	101	1239.90	143	359.95	86	445.73	59	189.30	83	1676.41	616	505.02
*F*		0.000		11.642		0.750		0.015		4.522		2.120		10.778
*p*		0.992		0.001		0.387		0.902		0.036		0.148		0.001
*One year*														
Male	99	34.24	50	46.48	107	38.53	65	58.35	51	26.23	48	63.56	420	41.29
Female	144	33.17	101	142.43	143	31.63	86	64.35	59	40.88	83	74.64	616	50.01
*F*		0.096		9.291		0.427		0.057		1.391		0.251		2.457
*p*		0.924		0.003		0.514		0.812		0.241		0.617		0.117

### Comparison of immunization effects in different age groups after vaccination

There were no significant differences in the anti-HBs seroconversion rate between the different age groups at 1 month after the third vaccination (*p* = 0.361, Fisher’s exact test), and all the six vaccines in different age groups achieved similarly high seroconversion rates: 99.5, 99.8, 98.8 and 100.0 %. However, the differences were statistically significant at 1 year after the third vaccination (*χ*^*2*^ = 11.182, *p* = 0.011), with positive rates of 84.7, 77.6, 72.1 and 75.6 %. Only in group III the differences between different age groups (15–24, 25–34, 35–44, 45–50) were statistically significant (*χ*^*2*^ = 10.044, *p* = 0.018) and further comparison found that the lower the age groups, the higher were the positive rates (*α* = 0.01, *r* = −0.176).

The anti-HBs GMTs in the four age groups at 1 month after the third vaccination for all six vaccines were 661.55, 478.63, 314.89 and 299.89 mIU/ml, and the differences were statistically significant (*F* = 7.545, *p* < 0.001). Thus, the differences at 1 year after the third vaccination were significant (*F* = 8.132, *p* < 0.001), with the anti-HBs GMTs of 75.33, 57.05, 30.27 and 35.59 mIU/ml. Further analysis based on the actual anti-HBs levels and the age found that the anti-HBs level was related to age at two time points: *r* = −0.153 (*α* = 0.01) 1 month after the full vaccination and *r* = −0.128 (*α* = 0.01) 1 year after the full vaccination, and the younger the age, the higher the anti-HBs level (Tables 4, 5).

**Table 4:** Anti-HBs GMT in different age groups after vaccination at two time points

Age group	Group I		Group II		Group III		Group IV		Group V		Group VI		Total	
	*n*	GMT	*n*	GMT	*n*	GMT	*n*	GMT	*n*	GMT	*n*	GMT	*n*	GMT
*One month*														
15~	37	276.00	19	1278.28	48	636.80	40	789.12	21	161.78	31	2711.17	196	661.55
25~	97	390.34	62	758.99	106	383.24	62	496.52	52	139.97	49	2276.57	428	478.63
35~	88	268.97	56	846.60	77	217.79	39	273.60	25	123.49	41	462.48	326	314.89
45~	21	190.45	14	1632.76	19	148.63	10	191.80	12	171.40	10	842.12	86	299.89
*F*		1.366		1.127		5.175		2.890		0.182		5.089		7.545
*p*		0.254		0.340		0.002		0.038		0.909		0.002		<0.001
*One year*														
15~	37	42.70	19	201.15	48	98.12	40	110.59	21	27.71	31	64.73	196	75.33
25~	97	48.10	62	96.95	106	38.25	62	66.59	52	55.05	49	82.90	428	57.05
35~	88	25.33	56	65.43	77	18.58	39	33.84	25	10.40	41	66.78	326	30.27
45~	21	13.74	14	201.46	19	16.49	10	38.72	12	58.46	10	50.68	86	35.59
*F*		2.005		1.868		5.935		1.641		4.889		0.287		8.132
*p*		0.114		0.138		0.001		0.182		0.003		0.834		<0.001

**Table 5:** A bivariate correlation on age and anti-HBs level for different vaccines

	Group I	Group II	Group III	Group IV	Group V	Group VI	Total
*r* (one month)	−0.060	0.034	−0.237^a^	−0.255^a^	0.013	−0.379^a^	−0.153^a^
*r* (one year)	−0.144	−0.081	−0.235^a^	−0.042	−0.012	0.073	−0.128^a^

^a^ *α* = 0.01

No spontaneous adverse effects related to the six vaccines were reported by the participants.

## Discussion

An American study reported that the 25- to 44-year-old age group showed the highest proportion of new hepatitis B infections [9], and once infected by hepatitis B, these patients’ children may be at high risk of hepatitis B infection because marriage and child-rearing are common in this age population [10, 11]. Hepatitis B vaccination could lead to a 38–47 % reduction in future HBV-related deaths [12]. Currently, the recommended hepatitis B vaccination dose abroad for healthy adults is 20 μg, and previous studies found that the immunological effect of 20 μg dose vaccination for adults was better than that of 10 μg dose vaccination [13–16]. The common healthy adult hepatitis B vaccine dose is 10 μg in China. We included both 10 μg dose vaccines and 20 μg dose vaccines in our study, and all six of the vaccines were administered to six groups: groups I–IV included four 10 μg dose vaccines (Shenzhen Kangtai, Dalian High-Tech, North China Pharmaceutical and GlaxoSmithKline, respectively), and groups V and VI included two 20 μg dose vaccines (North China Pharmaceutical and GlaxoSmithKline).

The six vaccines used in this study were administered intramuscularly to qualified participants, and immunogenicity was demonstrated in all participants; the seroconversion rates were obtained at 1 month and the seroreversion rates were obtained at 1 year after the third and final vaccination. Previous research has shown that, in general, hepatitis B vaccination programs (0-1-6 schedule) in healthy adults induce seroconversion rates of over 90 % [17]. In our study, the average seroconversion rate was 99.4 % and the anti-HBs GMT was 429.06 mIU/ml at 1 month after the third vaccination. The total positive rate was 77.0 % and the anti-HBs GMT was 47.36 mIU/ml at 1 year after the third vaccination. An anti-HBs level ≥10 mIU/mL is considered to confer vaccine-elicited protection against HBV infection [18, 19], and thus, all six hepatitis B vaccines in this study achieved good immunization effects at both time points, although there was an obvious decrease in the positive rate after 1 year. The decrease in anti-HBs protective rate depended upon the peak anti-HBs value [20]. A bivariate correlation between the anti-HBs level 1 month after the full vaccination and the anti-HBs level 1 year after the full vaccination found that the higher the anti-HBs level at 1 month was, the higher the anti-HBs level at 1 year was (*r* = 0.430). In the present study, the 10 μg dose vaccines obtained better immune seroconversion than the 20 μg dose vaccines at 1 month after the third vaccination. For the reason of large sample data, little difference may be tested when analyzed. According to the seroconversion rates 1 month after the full vaccination, both 10 μg dose vaccine and 20 μg dose vaccine could achieve good immune response. It was also shown that, in China, the 10 μg dose of hepatitis B vaccines could obtain good protective effects [21]. The differences in anti-HBs levels between different vaccines were significant at the two time points. The positive rate for the Dalian High-Tech vaccine (Lot Nos. 2009030906 and 2010010106; dose: 10 μg; Dalian High-Tech Biopharmaceutical Co., Ltd., China) was the highest among the four 10 μg dose vaccines at the 1 year time point after the third vaccination, while the seroconversion of subjects who received this vaccine was not significantly different at 1 month after the third vaccination, which may show that this vaccine has good stability. The positive rate of the GlaxoSmithKline vaccine (lot No. XHBVB554AA; dose: 20 μg; GlaxoSmithKline, UK) was significantly higher than another 20 μg dose vaccine at 1 year after the third vaccination, and this may also suggest that the vaccine has good stability.

In the present study, all six vaccines produced good immune effects for both genders and subjects of different ages. The anti-HBs GMTs for females were higher than that for males at 1 month after the third vaccination, and the anti-HBs levels showed a negative correlation with age 1 month after the full vaccination. These results indicate that the anti-HBs level attained after the third vaccination was related to age, which was consistent with previous studies [22]. Beside the independent effects of the three factors (age, vaccine and sex) on the anti-HBs levels, the interaction was found between age group and vaccine type 1 month after the full vaccination through further analysis (*F* = 1.942, *p* = 0.017), and younger age can contribute to the enhancement effect of vaccines. Apart from male gender and older age, well-known factors that lead to low hepatitis B vaccine immunogenicity include host-related obesity (including those who are non-obese but overweight), smoking, alcohol intake, chronic diseases (cirrhosis, diabetes, mellitus, chronic renal failure), immune suppression, genetic variation, injection site and storage conditions [23–32].

In our study, we selected six different vaccines and evaluated the immune effects at two time points. The immune effect of the 10 μg dose hepatitis B vaccine produced by Dalian High-Tech was better than that of the other three 10 μg dose vaccines, and the immune effect of the 20 μg dose vaccine produced by GlaxoSmithKline was better than the other 20 μg dose vaccine produced by the North China Pharmaceutical Company. Different counties where the same vaccine was used had the similar immune response (Table 6). The same vaccines vaccinated in other studies also achieved similar good antibody responses [33–36]. They produced high anti-HBs seroreversion/positive rates and anti-HBs GMTs, and persistent immune protection would be extended as a result of the high anti-HBs peak value [32]. The 10 μg dose of hepatitis B vaccine produced by Dalian High-Tech and the 20 μg dose vaccine produced by GlaxoSmithKline are recommended for vaccination of adults.

**Table 6:** Anti-HBs PSR and GMT of six vaccines separated by county at two time point

Vaccine group	County	*N*	PSR ( %)		*p*	GMT		*p*
			One month	One year		One month	One year	
I	1	97	100.0	82.5	0.026	248.66	38.22	0.014
	5	146	100.0	69.9		347.62	30.85	
II	2	151	100.0	85.4		906.07	98.30	
III	3	70	100.0	70.0	>0.05	328.32	36.43	0.004
	7	180	99.4	68.9		331.13	33.67	
IV	7	151	100.0	76.2		453.25	61.69	
V	4	110	99.1	76.4		142.98	33.28	
VI	6	131	96.9	87.8		1335.45	70.37	

There are some limitations of our study. First, the number of participants lost to follow-up was relatively large and we could not obtain the anti-HBs results in participants who were missing at 1 year, but the subjects who were lost follow-up had similar demographic characteristics as subjects who finished the one-year follow-up, and it is unlikely that a difference in the persistence of immunity would have occurred if less subjects had been lost to follow-up. In addition, further study is required to analyze the trend in seroreversion rates, and more time points such as 1 month after the first dose and the second dose should be individually analyzed.
